# Comparative evaluation of the effects of three hydraulic calcium silicate cements on odontoblastic differentiation of human dental pulp stem cells: an *in vitro* study

**DOI:** 10.1590/1678-7757-2022-0203

**Published:** 2022-11-04

**Authors:** Hadi ASSADIAN, Arash KHOJASTEH, Zahra EBRAHIMIAN, Fereshteh AHMADINEJAD, Helia Sadat Haeri BOROOJENI, Mahboubeh BOHLOULI, Mohammad Hossein NEKOOFAR, Paul MH DUMMER, Hanieh NOKHBATOLFOGHAHAEI

**Affiliations:** 1 Tehran University of Medical Sciences School of Dentistry Department of Endodontics Tehran Iran Tehran University of Medical Sciences, School of Dentistry, Department of Endodontics, Tehran, Iran.; 2 Shahid Beheshti University of Medical Sciences Research Institute of Dental Sciences Dental Research Center Tehran Iran Shahid Beheshti University of Medical Sciences, Research Institute of Dental Sciences, Dental Research Center, Tehran, Iran.; 3 Tehran Iran Dentist, private practice, Tehran, Iran.; 4 Shahrekord University of Medical Science Cellular and Molecular Research Center Shahrekord Iran Shahrekord University of Medical Science, Cellular and Molecular Research Center, Shahrekord, Iran.; 5 Shahid Beheshti University of Medical Sciences School of Advanced Technologies in Medicine Department of Tissue Engineering and Applied Cell Sciences Tehran Iran Shahid Beheshti University of Medical Sciences, School of Advanced Technologies in Medicine, Department of Tissue Engineering and Applied Cell Sciences, Tehran, Iran.; 6 Cardiff University College of Biomedical and Life Sciences School of Dentistry Cardiff UK Cardiff University, College of Biomedical and Life Sciences, School of Dentistry, Cardiff, UK.

**Keywords:** Somatic stem cell, Dental pulp cappings, Dentinogenesis

## Abstract

**Objective:**

The study aimed to compare the response of human dental pulp stem cells (hDPSCs) towards three hydraulic calcium silicate cements (HCSCs) by measuring cytotoxicity and expression of dentinogenic genes.

**Methodology:**

Dental pulps of five impacted mandibular third molars were extirpated as a source for hDPSCs. Next to culturing, hDPSCs were subjected to fluorescence-activated cell sorting after the third passage to validate stemness of the cells. Human DPSCs were exposed to diluted supernatants of OrthoMTA (OMTA), Biodentine (BD) and Calcium-Enriched Mixture (CEM) at concentrations 10, 25, 50 and 100% at the first, third and fifth day of culture. Then, cells were exposed to 10% concentrations supernatant of HCSCs to determine *DSPP* and *DMP1* gene expression, using a quantitative polymerase-chain reaction. Data were analyzed using one-way and three-way ANOVA, followed by *Tukey post hoc* statistical tests.

**Results:**

Optimal cell proliferation was observed in all groups, regardless of concentration and time-point. HCSC supernatants were non-cytotoxic to hDPSCs at all three time-points, except for 100% Biodentine on day five. On day seven, OMTA group significantly upregulated the expression of *DSPP* and *DMP1* genes. On day 14, expression of *DMP1* and *DSPP* genes were significantly higher in BD and OMTA groups, respectively.

**Conclusion:**

Biodentine significantly upregulated DMP1 gene expression over 14 days, whereas CEM was associated with only minimal expression of *DSPP* and *DMP1* .

## Introduction

Vital pulp treatments (VPT) are used to preserve pulpal integrity and function in immature permanent teeth. However, recent studies have focused on VPT use in mature teeth with carious pulpal exposures with partial irreversible pulpitis.^1^ Following successful VPTs, such as direct pulp capping (DPC), deposition and organization of hydroxyapatite crystals take place in the dentine-pulp complex, regulated by a collagen template and non-collagenous acidic proteins. Hydraulic calcium silicate cements (HCSCs) are used widely in VPT due to their impact on the formation of calcified bridges over exposed pulps.^2^ Upon proper choice of material in VPT, pulpal space is successfully sealed to hamper bacterial penetration and maintain remnant pulpal health following the formation of a calcified dentine-like bridge.^1^ Therefore, presence of viable human dental pulp stem cells (hDPSCs) enables a yielded respond, migration, proliferation and, eventually, differentiation into secretory cells for formation of this bridge **.** ^3^ The hDPSCs can be also applied in regenerative endodontic treatments (RETs), whose stem cells should remain viable after being exposed to HCSCs directly or indirectly to continue root formation.^4^ HDPSCs reside in specific niches and remain in their undifferentiated state as a reservoir within their microenvironment.^5^ Viable hDPSCs can differentiate into secretory odontoblast-like cells via regulative impacts of signaling molecules such as transforming growth factor-beta (TGF-β) superfamily including TGF-β1 and -3, fibroblast growth factor (FGF), bone morphogenetic proteins (BMPs) such as BMP-2, BMP-4, BMP-7, and heme oxygenase-1 enzyme.^5^ The PI3K/AKT/mTOR signaling pathway modulates the proliferation of hDPSCs in a sequential fashion, mediated by growth factors, cytokines and environmental stimuli.^5^

Members of the Small Integrin-Binding Ligand N-linked Glycoprotein (SIBLING) family such as dentin matrix protein-1 ( *DMP1* ), osteopontin (OPN), matrix extracellular phosphoglycoprotein (MEPE), bone sialoprotein (BSP), and dentine sialophosphoprotein (DSPP) are associated with dentine bio-mineralization.^6^ The hDPSCs, which can be detected by quantitative reverse transcriptase polymerase chain reaction (RT-PCR) and immunofluorescence, can represent markers of odontoblastic differentiation, including those of the non-collagenous acidic proteins. Dentine sialophosphoprotein (DSPP) is present in bone, dentine, cementum and, to a much lower extent, in some non-mineralized tissues. The *DSPP* gene expression in dentine is greater than that in non-dental tissues. The cleaved products of *DSPP* gene are dentine phosphoprotein (DPP) and dentine sialoprotein (DSP), resulting in formation of active fragments from an inactive precursor that allows spatiotemporally controlled mineralization of dentine.^7^ DPP is a chief promoter of the biomineralization process via hydroxyapatite (HA) crystals formation. This is assumed to result from established electrical affinity between negatively charged portions of DPP and positively charged calcium, making it easier for Ca^++^ ions to be presented to collagen fibers located in the mineralization front.^7^

Abundant in peritubular dentine, *DMP1* is another member of the SIBLING family and an important component of the non-collagenous dentine matrix.^6^ During normal and pathological dentine formation, *DMP1* can induce differentiation of hDPSCs into odontoblast-like cells and regulate dentine mineralization and organization of the collagenous matrix.^8^ In carious lesions, *DMP1* secretion is a consequence of mediated dentine softening by acidogenic bacterial activity. The secretion causes differentiation of pulpal cells into odontoblasts.^9^ The *DMP1* expression can influence intra- and extra-cellular mineralization, and acidic domains of *DMP1* in the extracellular matrix can initiate nucleation of hydroxyapatite crystals. However, we detected *DMP1* -expressed proteins within nucleus during the initial phases of odontoblast differentiation. Distinctive similarities of *DMP1* and *DSPP* — especially in terms of tissue localization, proteolytic processing, and high molecular weight forms — indicate their synergistic function and possible physical interactions.^7^ Similar to *DSPP* , acidic residues of *DMP1* can regulate odontoblastic differentiation *in vitro* and dentinogenesis *in vivo* , and act as extra-cellular nuclei for development of HA crystals.^10^

Several commercially available HCSCs introduce promising results for VPTs. Biodentine^TM^ (Septodont, Saint-Maur-des-Fossès, France) is a member of the HCSCs family with shortened span of setting time and improved physical and handling features.^11^ Upon direct pulpal contact, Biodentine, considered a reference material to compare newer HCSCs^12^ , significantly enhances cellular proliferation, migration, attachment, and reparative dentinogenesis.

Calcium-Enriched Mixture^®^ (CEM; BioniqueDent, Tehran, Iran) is another HCSC, composed of several calcium compounds such as calcium-sulfate, -phosphate, -carbonate and -silicate.^11^ CEM has promising sealing and antimicrobial properties. Moreover, CEM reports a clinically high (>90%) success rate in comparison with tooth-colored ProRoot MTA (Dentsply Sirona, Tulsa Dental, Tulsa, OK, USA), in a multi-centered clinical trial, involving pulpotomy of mature molars with partial irreversible pulpitis.^13^

OrthoMTA^®^ (OMTA; BioMTA, Soul, Daejeon, Korea) is a HCSC proposed for VPT and root-end filling. OMTA can form an interfacial hydroxyapatite layer and its small particle size allows penetration into dentinal tubules for a better sealing. Since it has a low content of heavy metals, OMTA is a safe HCSC, which demonstrates short-term cytotoxicity in MG-63 cells comparable to ProRoot MTA, and upregulates dentine sialophophoprotein ( *DSPP* ) gene expression in MDPC23 cells *in vitro* .^14^ HCSCs used for RETs/VPTs should be biologically inert or induce repair while interacting with hDPSCs. Therefore, cell-material compatibility for these constructive phenomena is essential^15^ Biocompatibility of HCSCs is as important as preventive sealing characteristics against bacterial leakage in determining the favorable procedure prognosis. The traditional use of calcium hydroxide (CH) is becoming obsolete for VPTs, mainly due to the porous dentinal bridges formed, poor dentinal adhesion and compromised resistance against bacterial microleakage. Alternatively, HCSCs are becoming standard of care in VPT/RET.^16^ Although incompletely understood, HCSCs lead to comparable hDPSC-mediated hard tissue formation results, with calcium hydroxide. Fewer inflammatory and necrotic changes and a more rapid, homogenous and predictable dentine-like barrier is expected with HCSCs.^16^ In this study, we selected HCSCs including Biodentine, OMTA and CEM, due to their apt clinical performance in VPTs/RETs.^17^ However, limited data exists regarding the active viability of hDPSCs and their dentinogenic differentiation induced by various brands of HCSCs. Since comparative information about the biological effects of CEM and OMTA regarding Biodentine is scarce, this study aimed to compare the effect of these HCSCs on cell proliferation and expression of *DSPP* and *DMP1* genes as markers of dentinogenesis by hDPSCs *in vitro* .

## Methodology

The manuscript of this laboratory study has been written according to Preferred Reporting Items for Laboratory studies in Endodontology (PRILE) 2021 guidelines.

### Isolation of human dental pulp stem cells (hDPSCs)

**Figure 1:**
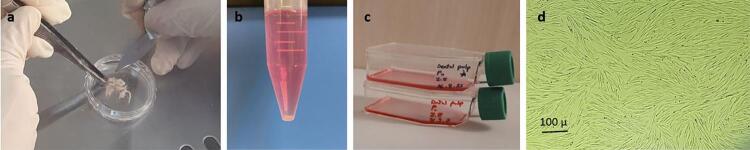
a. Pulpal tissues were rinsed by penicillin/streptomycin-containing phosphate-buffer solution (PBS) and sectioned into several pieces. Pulpal tissues were placed in collagenase I solution for 30 minutes and incubated at 37ºC and 5% CO2 with 100% humidity. Then, to neutralize collagenase solution, standard culture solution containing high-glucose DMEM, penicillin/streptomycin, 15% fetal bovine saline and fungizone were added and centrifuged at 1500 rpm for 10 minutes. b. Centrifuged to obtain cellular pellet in the falcon. c, d. The isolated cells were transferred into culture flasks containing standard medium

Five impacted or caries-free erupted third molar teeth were extracted from 3 systemically healthy patients from 20 to 30 years old. The teeth were placed immediately in high-glucose Dulbecco’s Modified Eagle Medium (DMEM) and penicillin/streptomycin (ThermoFisher Scientific, Waltham, MA, USA) that contains the buffer solution. Pulps of the teeth were extirpated under sterile laboratory conditions, rinsed using phosphate-buffered solution (PBS) medium and then sectioned into several pieces. The pulpal tissues were placed in collagenase I solution for 30 minutes and incubated at 37ºC and 5% CO_2_ with 100% humidity. To neutralize the collagenase solution, a standard culture solution containing high-glucose DMEM, penicillin/streptomycin, 15% fetal bovine saline and fungi-zone was added and centrifuged at 453 g for 10 minutes. Then, the resultant solution was added to a T25 flask containing standard culture medium and incubated ( Figure 1 ). After the third passage, the cells were evaluated for surface markers such as CD90, CD73, CD105, CD45, and CD34 using flow cytometry.

### Flow cytometry analysis

Fluorescence-activated cell sorting (FACS) evaluation was performed using standard protocols and measurement criteria to confirm the expression of surface markers, including CD90, CD73 and CD105 and the absence of CD45 and CD34 markers. Monoclonal fluorescein isotyocyanate and phycoerythrin-conjugated antibodies were placed on the isolated cells. Anti-CD90-FITC, anti-CD73-FITC, anti-CD105-PE, anti-CD45-FITC and anti-CD34-PE at a concentration of 2 μg/mL were used. Labeled cells were then evaluated using flow cytometry (FACS Calibur; BD Biosciences, San Jose, CA, USA) and standard flow cytometry files were analyzed using FlowJo 7.6.1 software (FlowJo LLC, Ashland, OR, USA). Samples containing at least 90% of fluorescent labeled cells were considered positive (n=3).

### Sample preparation

All HCSCs were prepared according to the manufacturers’ instructions described below:

Biodentine (BD) capsules were selected and gently tapped on a hard surface to loosen the powder. Then, five drops from the liquid container were introduced into the capsule and mixed with an amalgamator (Ultramat Amalgamator; Southern Dental Industries, Bayswater, Victoria, Australia) at a speed of 4000-4200 rpm for 30 seconds.^18^

OMTA vials were opened and mixed with distilled water. With each cap closed, the vial was mixed for 15 seconds using the centrifuge provided by the manufacturer. Then, each vial was re-opened, excess water removed and the mixed material transferred using the carrier provided.^19^

CEM was prepared according to the developer’s early publication^20^ in a powder-to-liquid ratio of 3:1 to form a putty consistency.

A standardized 0.2 cm^3^ increment of each cement was transferred to a 12-well plate using minimal pressure to homogenously cover the bottom of each well. The samples were incubated for 24 h at 37ºC and 5% CO2 with 100% humidity, and sterilized by gamma irradiation with an intensity of 37 kGy, activity of 6820 Ci and dose rate of 1.62 Gy/sec. Finally, 3 mL high-glucose DMEM was added and incubated at 37ºC and 5% CO2 with 100% humidity for another 24 h. The samples were prepared so that the exposed cement surface to liquid ratio was established at 126.6 mm^2^ /mL according to ISO standards.^21^

After 24 h, the supernatant was removed and filtered using a 0.45-size filter and 10, 25, 50 and 100% concentrations were used for the subsequent cellular viability tests to determine the most appropriate dilution for the real-time polymerase chain reaction (RT-PCR) (n=3).

### Cell proliferation assay

Initially, 10^4^ cells were counted using hemocytometer and added to 48-well plates with 500 µL of the standard culture medium for ٢٤ h of incubation. Afterwards, the supernatants of the test materials with 10, 25, and 50% dilutions were added to the plates and incubated in triplicate. A 3- (4,5-di-2-yl)-2,5-ditetrazolium bromide (MTT) solution was mixed with the culture medium in a 1:9 ratio to result in a homogenous solution. The culture medium was emptied in plate wells and 100 µL of the prepared MTT solution was added to each plate and incubated for 4 h. Then, the culture medium was removed completely from the plate wells and 100 µL DMSO solution was added to each well, then after five minutes it was added to a 96-well plate. Absorption of each well, containing either the sample or blank, was analyzed at a wavelength of 570 nm by an ELISA plate reader (PowerWave^TM^ X52; BioTek Instruments Inc., Potton, UK). The MTT assay was carried out on days 1, 3, and 5 (n=3).

### Real-time reverse transcryptase polymerase chain reaction (RT-PCR)

The expression of dentin matrix protein-1 ( *DMP1)* and dentine sialophosphoprotein ( *DSPP* ) genes in hDPSCs was evaluated for odontogenic differentiation capacity, using Real-Time RT-PCR. Total RNA was extracted from the cells via RNX-Plus (Sinaclon, Tehran, Iran) reagent, following the manufacturer’s instruction. cDNA was synthezed using ReverAid First Strand cDNA Synthesis kit (Sinaclon, Tehran, Iran) and subjected to Real-Time PCR (StepOnePlus Real-Time PCR System; Applied Biosystems, Foster City, CA, USA) by a SYBR Green Master Mix Kit high ROX (Ampliqon; Odense, Denmark). The expression of Glyceraldehyde-3phosphate dehydrogenase gene was analyzed as an endogenous control. The cycle threshold (CT) value was normalized to endogenous control to estimate ΔCT. Mean fold change was estimated with the ‘Livak’ method using its control and the hDPSCs were cultured in the standard medium before treated with the supernatant mediums. Primers used for Real-Time-PCR analysis are presented in detail in Table 1 .

**Table 1:** Odontogenic markers' primer

Genes	Sequences	Tm	Product size
DSPP-F	GCCATTCCAGTTCCTCAAAGCA	61,04	199
DSPP-R	TCCCTTCTCCCTTGTGACCAT	60,49	199
DMP1-F	AATTCTTTGTGAACTACGGAGGGTA	59,99	194
DMP1-R	ATGACTCACTGCTCTCCAAGG	59,45	194
GAPDH -F	CTCTCTGCTCCTCCTGTTCG	59,54	114
GAPDH -R	ACGACCAAATCCGTTGACTC	58,21	114

### Statistical analysis

Statistical analysis of data was performed with SPSS software (IBM SPSS, Version 25; IBM, NY, USA) and P values of less than 0.05 were defined as statistically significant. Comparison of cell proliferation at different times, concentrations and with different test materials, as well as the fold changes in expression of *DSPP* and *DMP1* genes between and among test materials in comparison with the control were analyzed using three-way and one-way ANOVA followed by *Tukey post hoc* statistical tests (n=3).

## Results

### Results of the flow-cytometric analysis

**Figure 3:**
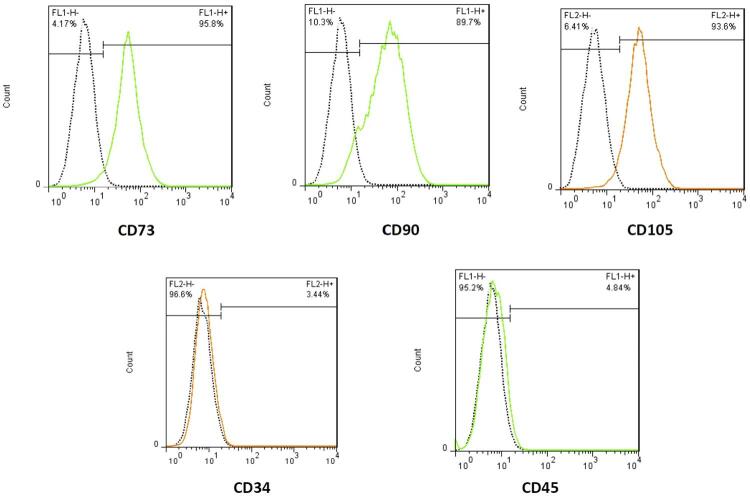
Flow cytometric characterization of hDPSCs. hDPSCs positively expressed CD73, CD90, and CD105 and did not express CD34 and CD45

Regarding stemness markers expression and differentiation capability, we evaluated fibroblast-like cells derived from hDPCSs at their third passage. The cells had positive expressions for CD73 (95.8%), CD90 (89.7%) and CD105 (93.6%) and negative expressions for CD34 (96.6%) and CD45 (95.2%) ( Figure 3 ).

### Results of MTT assay

**Figure 4:**
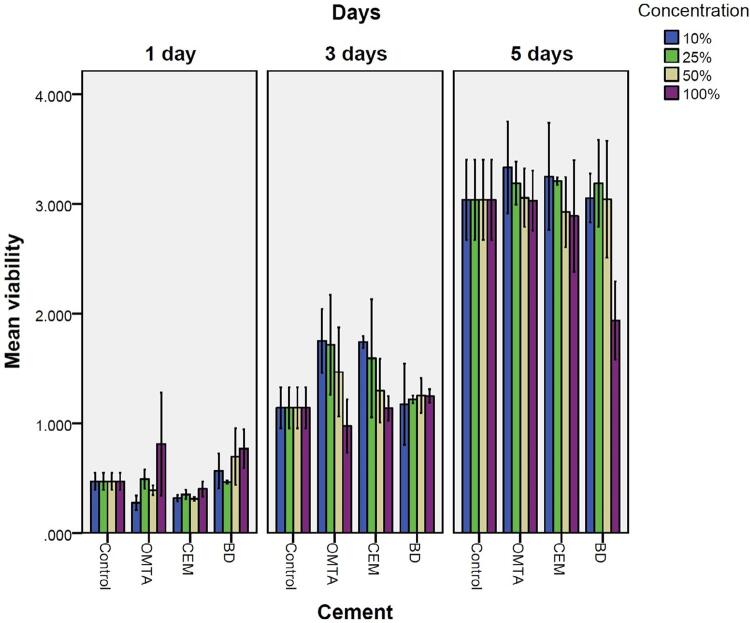
MTT assay. The one-way ANOVA test showed no significant difference in cell proliferation after exposure to 10, 25, 50, and 100% concentrations OMTA and CEM cement in days one, three and five. (p>0.05) Furthermore, no significant difference was observed in cell proliferation following exposure to different concentrations of BD in first and third days (p>0.05). The test demonstrated a significant decrease in cell proliferation following exposure to 100% concentration of BD (p=0.012). Three-way ANOVA test showed that cell proliferation of test materials in different concentrations presented no significant difference in days one and three compared to control group (p>0.05). On the other hand, cell proliferation was significantly lower than control on day five when exposed to 100% concentration of BD (p=0.016)

Based on the one-way ANOVA test, we observed an insignificant difference in cell proliferation after exposure to 10, 25, 50, and 100% concentrations OMTA and CEM cement in first, third, and fifth days (p>0.05). Furthermore, we identified an insignificant difference in cell proliferation following exposure to different concentrations of BD in first and third days (p>0.05). However, the test presents a significant decrease in cell proliferation, following exposure to 100% concentration of BD (p=0.012). Three-way ANOVA test showed that cell proliferation of test materials in different concentrations had an insignificant difference in days 1 and 3 compared to control group (p>0.05). Contrarily, when exposed to 100% concentration of BD cell proliferation was significantly lower than control on day five (p=0.016) ( Figure 4 ).

### Real-time PCR test results

#### Results on day seven

**Figure 2:**
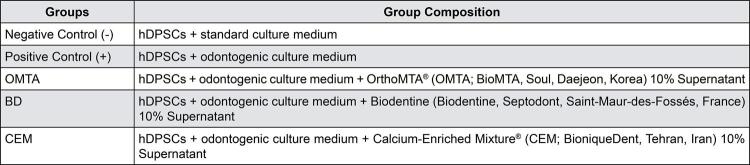
Group Substances

**Figure 5:**
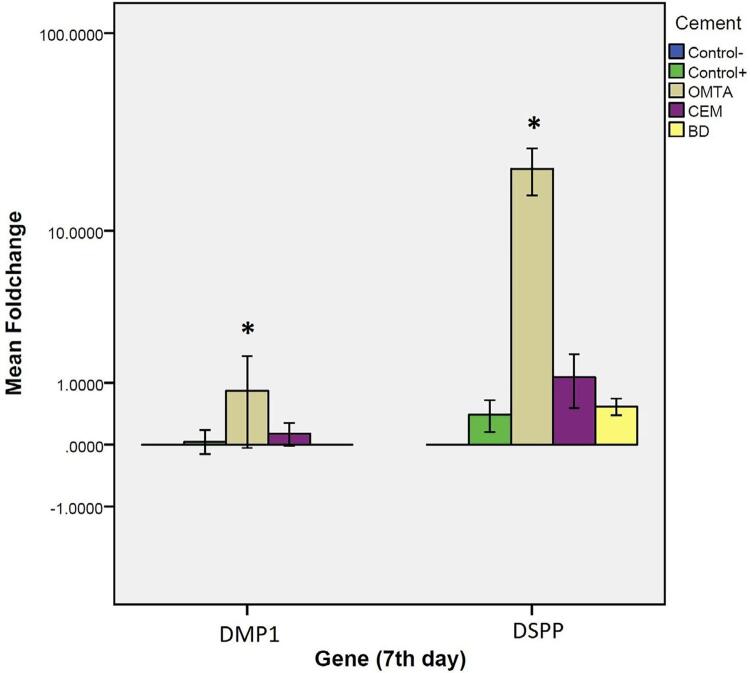
Real-time PCR test results comparing gene expression of DSPP and DMP1 induced by the test materials on day seven. One-way ANOVA revealed a statistically significant difference in expression of DSPP and DMP1 genes between test materials on day seven. Complementary Tukey HSD test revealed that OMTA significantly increase expression of the aforementioned genes (p=0.0002)

We verified a significant difference in *DSPP* and *DMP1* genes expression between genes expression between test materials ( Figure 2 ) on day seven. The complementary *Tukey HSD* test revealed that OMTA significantly increased expression of the aforementioned genes (p=0.0002) ( Figure 5 ).

#### Results on day 14

**Figure 6:**
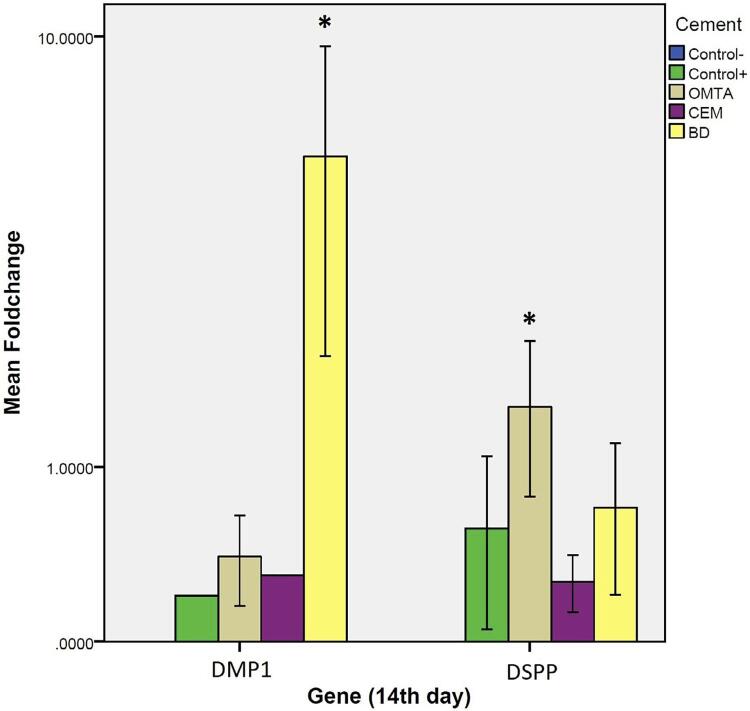
Real-time PCR test results comparing gene expression of DSPP and DMP1 induced by the test materials on day 14. One-way ANOVA test indicated a significant difference in expression of DSPP and DMP1 genes between test materials on day 14. Tukey HSD test indicated that BD significantly increase expression of DMP1 and OMTA significantly increased DSPP gene expression (p=0.0003)

One-way ANOVA test indicated a significant difference in expression of *DSPP* and *DMP1* genes between test materials on day 14. The *Tukey HSD* test indicated that Biodentine significantly increased *DMP1* expression (p<0.001) and OMTA significantly increased *DSPP* gene expression (p=0.0003) ( Figure 6 ).

## Discussion

Regarding cytotoxicity, cell proliferation and expression of dentinogenesis markers, *DSPP* and *DMP1* genes, we aimed to compare the *in vitro* hDPSCs response towards application of OMTA, BD and CEM, in 10, 25, 50 and 100% concentrations. The supernatants of OMTA, CEM and Biodentine were non-cytotoxic to hDPSCs at all time-points except for 100% Biodentine on day five.

OMTA and CEM differed insignificantly regarding cyto-compatibility in all the time intervals tested. Due to the lack of data, these results cannot be compared with any other study. However, several authors independently reported the promising biocompatibility of OMTA and CEM, mainly in comparison with other HCSCs. For example, CEM had similar cytotoxicity and genotoxicity to MTA on cultured L929 fibroblasts in fresh and set states in two separate studies.^22 , 23^ Pornamazeh, et al.^24^ (2017) compared CEM with RetroMTA (BioMTA, Seoul, Korea), produced by the same manufacturer as OMTA, on mouse L929 fibroblasts. The authors stated that the viability of the exposed cells was quite similar in fresh and set CEM in comparison with RetroMTA. The statement partially agrees with the results we obtained, although structural and compositional differences between OMTA and RetroMTA should be observed as well. RetroMTA consists of hydrophilic materials, underived from Portland cement including silicon oxide, calcium carbonate, aluminum oxide, and hydraulic calcium zirconia complex, which inhibits discoloration and is suitable for aesthetically involved treatments.^24^ The similar cyto-compatibility of CEM and MTA using hDPSCs has been reported.^25^ Although the detailed information about the composition of the MTA used in that study was suppressed, the authors reported an initial decrease in cell viability, which was not observed in this study. We can attribute this to the direct material-cell contact in their study, setup in comparison with the use of supernatants in our study. In another similar study, CEM had equally favorable compatibility with hDPSCs in comparison with other formulations of MTA including ProRoot MTA (PMTA; Dentsply Sirona), Angelus MTA (AMTA; Angelus, Londrina, Parana, Brazil), and Root MTA (RMTA; Lotfi research group, Tabriz, Iran).^26^ Moreover, similarities between CEM- and MTA-mediated cellular responses have also been reported in other cell sources such as human gingival fibroblasts and L929 cells.^24 , 27^ The presence of mineral deposits from dental pulp stem cells of human exfoliated primary teeth by CEM is further evidence of its favorable cell-cement interaction. A similarity between OMTA and gray ProRootMTA (Dentsply-Tulsa Dental Specialties, Johnson City, OK, USA) has been reported in terms of chemical composition.^28^ On the other hand, the impossibility of detailed comparison of various MTA preparations and the demand of cautiously interpreted investigations using different materials cannot be overestimated. Since the investigation on OMTA is scarce, we only mentioned the results of studies on ProRoot MTA due to the their chemical and structural similarities.

In this study, Biodentine mediated cellular viability was insignificantly different on days 1 and 3. However, on day five the concentration of cell proliferation decreased at 100%. Luo, et al.^29^ (2014) reported corresponding results, which demonstrated that greater Biodentine concentrations can reduce hDPSC proliferation. Likewise, Zanini, et al.^30^ (2012) evaluated 1, 2, and 4 mg/mL concentrations of Biodentine to determine the effect of material concentration on cell viability in their pilot study, finding that the lowest concentration was the most suitable for further experiments. The authors also reported an initially decreased bio-tolerance of Biodentine on immortalized murine pulp cells.^30^

After exposure to 10% and 100% dilutions, the results showed that Biodentine was associated with a significantly lower number of proliferative rate of viable cells compared to OMTA and CEM. A comparison with the same materials on the same cellular population has not been reported. However, Biodentine had an almost similar biocompatibility with different brands of MTA on hDPSCs; stem cells of the apical papilla; bone tissue; murine odontoblast-like cells (MDPC-23); L929 mouse fibroblasts; human pulp fibroblasts; osteoclasts derived from murine bone marrow macrophages; human monocytes; and animal (rat) models. Biodentine is also a reference material for investigating newly introduced HCSCs.^12^ Nevertheless, Biodentine-induced toxic or inflammatory reactions have been reported on human periodontal ligament stem cells; stem cells from exfoliated primary teeth; apical papilla cells; and 3T3 fibroblasts, which corroborates the results of the present investigation. At the same time, in a histologic evaluation, more collagen-rich fibrous capsule formation is reported in response to Biodentine implants in comparison with MTA.^31^ Although the exact mechanism in which a HCSC interacts with its surrounding cells/tissue is not understood thoroughly, the resultant alkalinity of HCSC following setting and CH release from the materials are responsible for the decrease in cell viability, whereas we can attribute their viability effects to the presence of calcium and silicon ions probably in the form of tricalcium silicate. Additionally, the presence of calcium chloride in Biodentine liquid was pointed out as a cause for its greater initial toxicity.^32^ Another possible explanation for the diversity in the results of bio-tolerance studies can be the direct versus indirect method of exposure of the test material with the target cells, which may result in a more intense cellular damage, due probably to the surface roughness of the material, which was evident in the unpublished pilot studies of this investigation.

The gene expression of *DSPP* and *DMP1* was significantly greater in OMTA compared to the other materials on day seven. On day 14, expression of *DMP1* in Biodentine and *DSPP* in OMTA was significantly greater than other groups. The MTA (White MTA; Angelus) resulted in elevated expression of *DMP1* on day seven and Biodentine from day 14 onwards.^3^ By using ProRoot MTA (Dentsply Tulsa Dental Specialties, Tulsa, OK, USA)^33^ , we observed an improved expression of odontoblastic differentiation genes such as osteocalcin ( *OCN* ), bone sialoprotein ( *BSP* ), type I collagen ( *COL1* ), *DSPP* , alkaline phosphatase ( *alp* ), and *DMP1* . We associated Biodentine and OMTA with a greater expression of *DSPP* even when compared with the positive control group on day 14. Moreover, the study that reported increased *DSPP* expression in comparison with control in the same time interval (day 14)^21^ supported our investigation. We can mainly attribute the differences in odontogenic gene expressions by different HCSCs to the differences in the constituents of the materials. During setting of HCSCs, hydration of the calcium silicate content resulted in formation of calcium hydroxide, which produces Ca^++^ ions within the surrounding environment. The production can be a major promoter for bioactive reactions in the adjacent tissues and cells. Although Biodentine-induced differentiation of hDPSCs occurs via ERK1/2, JNK MAPK and CaMKII pathways, in general, the exact mechanism in which stimulation of dentinogenic markers by HCSC takes place is unknown.

In this study, OMTA had greater expression of *DSPP* and *DMP1* genes compared to CEM and positive and negative control groups. However, Asgary, et al.^34^ (2014) reported that the expression of *DSPP* and *DMP1* showed no significant difference between and among MTA, CEM and differentiation medium, although the expression was higher than growth medium used as the control. This can be attributable to the difference in exposure of the test material with the cells. Presumably, direct contact of CEM could result in its improved odontogenic profile.

The effect of calcium silicate-based materials on hDPSCs has been evaluated frequently, increasing the conclusions due to different constituents of the materials tested; methodologic details such as diversities in assessments; acquisition of material extracts; direct versus indirect contact of the test material with cellular medium, etc. In this study, we used 10% dilution of the test material extracts for evaluation of *DSPP* and *DMP1* gene expressions.

If cells had direct contact with experimental cements, as stated by Asgary, et al.^34^ (2014), we could have had more clinically relevant results. However, extensive cellular necrosis in the direct contact model prohibited subsequent evaluations in the pilot study; therefore, we considered cement supernatant at various concentrations for further evaluations. Moreover, monolayer culture of hDPSCs bears drawbacks when compared with three-dimensional culture models that more suitably mimic clinical conditions. Three-dimensional culturing of the hDPSCs would have enabled spatiotemporal interactions among the neighboring cells and the extracellular matrix, providing a better foundation for cell proliferation and promoted differentiation. Regarding HCSCs, we used Biodentine, OMTA and CEM. Several studies indicating favorable biological effects of Biodentine exist in the literature, whereas comparable studies concerning OMTA and CEM are scarce. In most of the previous investigations,^35 , 36^ CEM provides similar biological behavior compared to MTA, whereas this result is partly questioned in the present study. We concluded that the supernatants of OMTA, CEM and Biodentine were non-cytotoxic to hDPSCs at all time-points, except for 100% Biodentine on day five. Biodentine upregulated *DMP1* gene expression significantly over a longer interval (14 day), whereas CEM was associated with minimal expression of these dentinogenic markers *in vitro* .
